# Body composition in Indian children and adolescents with type 1 diabetes

**DOI:** 10.1186/1687-9856-2015-S1-P18

**Published:** 2015-04-28

**Authors:** Vaman Khadilkar, Lavanya Parthasarathy, Anuradha Khadilkar, Shashi Chiplonkar

**Affiliations:** 1 Hirabai Cowasji Jehangir Medical Research Institute, Jehangir Hospital, Pune, Maharashtra, India

## 

Studies suggest that children and adolescents with type 1 diabetes (T1DM) have suboptimal body composition with higher fat mass and lower bone mass. Aim of our study was to compare body composition of Indian children with type 1 diabetes with age gender matched healthy controls.

In a cross-sectional study, body composition parameters were measured by DXA (Lunar DPX PRO, Total Body Densitometer) in 160 (74 boys) children with T1DM (attending type 1 diabetes clinic) and age gender matched healthy controls. Z scores for bone mineral content (TBBMC) for age, bone area for age (TBBA), TBBMC for TBBA, TBBA for height, lean body mass (LBM) for height, TBBMC for LBM[1] and fat and lean mass [2] were computed using Indian reference data. Anthropometry and tanner staging (TS) was assessed for all children. The height (HAZ), weight (WAZ) and BMI (BAZ) were converted to Z scores using contemporary Indian references [3].

Mean ages of boys and girls were 11.4±3.3y and 10.9±3.4y respectively. For both genders HAZ (boys -0.5 vs 0.3, girls -0.5 vs 0.1) and WAZ (boys -0.6 vs 0.0 and girls -0.5 vs -0.1) were significantly lower in diabetics, though BAZ scores were comparable. Similarly, mean Z scores were significantly lower for fat mass (boys -0.1 vs 0.2, girls -0.1 vs 0.1) and higher for lean mass (boys -0.4 vs -1.1, girls -0.3 vs -0.6) in diabetics for both genders. Diabetic boys and girls had lower android fat percent (boys 20 vs 25, girls 28 vs 32, P<0.05) when compared with controls. Mean Z scores for bone parameters showed TBBA for age (boys -0.1 vs 0.5, girls -0.1 vs 0.3), TBBMC for age (boys -0.1 vs 0.6, girls -0.1 vs 0.3) and TBBMC for LBM (boys -0.4 vs 0.0, girls -0.5 vs -0.2) were significantly lower in diabetics while all other bone parameters were comparable.

Indian children with type 1 diabetes had lower fat mass (including android fat) and higher muscle mass than controls; however, diabetics need special attention to optimize their bone health.
